# Intrinsic differences between males and females determine sex-specific consequences of inbreeding

**DOI:** 10.1186/s12862-016-0604-5

**Published:** 2016-02-09

**Authors:** Emily R. Ebel, Patrick C. Phillips

**Affiliations:** Institute of Ecology and Evolution and Department of Biology, 5289 University of Oregon, 97403 Eugene, Oregon USA; Present address: Department of Biology, Stanford University, 371 Serra Mall, Stanford, CA 94305 USA

## Abstract

**Background:**

Inbreeding increases homozygosity and exposes deleterious recessive alleles, generally decreasing the fitness of inbred individuals. Interestingly, males and females are usually affected differently by inbreeding, though the more vulnerable sex depends on the species and trait measured.

**Results:**

We used the soil-dwelling nematode *Caenorhabditis remanei* to examine sex-specific inbreeding depression across nine lineages, five levels of inbreeding, and hundreds of thousands of progeny. Female nematodes consistently suffered greater fitness losses than their male counterparts, especially at high levels of inbreeding.

**Conclusions:**

These results suggest that females experience stronger selection on genes contributing to reproductive traits. Inbreeding depression in males may be further reduced by sex chromosome hemizygosity, which affects the dominance of some mutations, as well as by the absence of sexual selection. Determining the relative contributions of sex-specific expression, genes on the sex chromosomes, and the environment they are filtered through—including opportunities for sexual selection—may explain the frequent though inconsistent records of sex differences in inbreeding depression, along with their implications for conservation and the evolution of mating systems.

## Background

Inbreeding depression, or the fitness decline resulting from mating among close relatives, has long been thought to underpin many important evolutionary processes [1]. Since inbreeding increases homozygosity, it exposes deleterious recessive alleles and eliminates any potential heterozygote advantage [2]. Inbreeding depression has been observed across many taxa [2–4] and has wide-ranging implications, particularly for the evolution of dispersal behavior, self-incompatibility, and other mating systems characters [2, 5]. However, considerable variation exists in the strength of inbreeding depression, especially among populations and environments and between sexes [6–11].

Most studies that have examined inbreeding depression in males and females independently have found one sex to be more “sensitive” to inbreeding than the other (Table 1). This sex-specificity has a direct bearing on conservation strategies for small populations [12, 13] and could contribute to the evolution of selfing and polyandry, which may be heavily influenced by inbreeding depression [14, 15]. Despite these potential consequences, it is difficult to predict which sex will be more vulnerable to inbreeding. Previous studies have found conflicting results that seem dependent on the study system and experimental conditions (Table 1), exposing the lack of a comprehensive framework for understanding sex-specific inbreeding depression and its evolutionary consequences.

**Table 1:** Summary of previous studies on sex-specific inbreeding depression. Only studies that quantified inbreeding depression comparably for both sexes are included

Species	Vulnerable sex	Traits examined	Conclusions	Reference
Wild gourd	Female	Fruit and flower number, seed germination, pollen success	Female function requires more resources than male function	[53]
Morning glory	Female	Flower and seed number, survival	Different numbers of loci affecting fitness or different average contributions	[67]
*Drosophila*	Male	Larval-adult survival, female fecundity, male mating success	Inclusion of the opportunity for male-male competition increases differences in inbreeding depression among sexes	[11]
*Drosophila*	Male	Egg hatchability, larval-adult survival, female fecundity, male mating success	Sexual selection makes inbreeding more costly for males	[7]
*Drosophila*	Both	Egg-to-adult viability	No sex-specific inbreeding depression	[68]
Stalk-eyed flies	Male/Both	Eyespan, thorax length, wing length	Sexually-selected trait (eyespan) more sensitive in males; no sex-specific differences in other traits	[69]
Beetle	Female	Adult mortality	Sex-specific alleles involved in inbreeding depression; hemizygosity causes male-specific selection	[10]
Beetle	Male	Sexual odorant signaling	Odorant may be male-only sexually selected trait	[70]
Beetle	Male	Proportion of offspring in competitive environment	Males have greater reproductive variance; stressful environment amplifies inbreeding depression in males	[71]
Butterfly	Male	Fertility	Sex-specific alleles involved in inbreeding depression; “direct or indirect fertility selection…operating differentially among the sexes”	[72]
Hihi (bird)	Male*	Embryo/nestling mortality	Size dimorphism may increase inbreeding sensitivity; *lack of comparably inbred females may suggest elevated mortality	[73]
Takahe (bird)	Female	Fledgling success	Sex-effect explanation “currently unknown”	[74]
Song sparrow	Female	Offspring number and survival	Maternal effects increase female inbreeding sensitivity; reproductive ecology may decrease male sensitivity	[54]
Song sparrow	Female/ Male/Both	Immune response (3 types)	“not clear why inbreeding effects should differ between males and females”; perhaps sex-specific variation in life-history allocation or physiology	[75]
Japanese quail	Female	Hatching success, viability, fertility	Maternal effects; delay of sexual maturity in females	[52]
Great tit	Both	Hatching, fledging, and breeding success	No sex-specific inbreeding depression	[76]
Great tit	Female	Hatching success	Maternal effects increase female inbreeding sensitivity	[51]
Zebra finch	Both/Female	Body mass, tarsus length, wing length, fat	Most morphological traits show similar patterns of inbreeding depression; sex-specific traits also vunerable	[77]
Mouse	Male	Adult and offspring survivorship, male competitive ability	Sexual selection makes inbreeding more costly for males	[58]
Gazelle	Female	Longevity	Longer female lifespan is more sensitive to inbreeding depression	[78]

In theory, the same mutations may affect inbred males and females differently if they vary between the sexes in patterns of dominance or intensity of selection [16]. Generally, little is known about how dominance differs between sexes or across the genome [16–18], with the exception of genes on the sex chromosome. In taxa with heteromorphic sex chromosomes, genes may be recessive for the sex with two copies (e.g. XX females), but necessarily dominant for the sex with a single copy (e.g. XY males), which could systematically influence inbreeding depression between the sexes.

Selection on the same variant could also differ between sexes due to differences in expression, life history, sexual selection, or a number of other factors [19–21]. While differential gene expression and the relative rate of evolution of genes related to sex-specific traits has become a central feature of studies of molecular evolution [22, 23], the relationship between these patterns and sex-specific inbreeding depression has remained largely unexplored. In the most extreme case, loci that have evolved completely sex-limited expression will be influenced only by selection acting on that particular sex, as may be the case for maternal effects [24–26] and sexually selected ornaments or behaviors [27, 28]. Many mutations are expressed in both sexes and have correlated effects in males and females [29], but may still have different fitness consequences that depend on behavior or the environment. For example, paralyzing *unc* mutations have stronger reproductive consequences for male than hermaphroditic *Caenorhabditis elegans* [30].

**Fig. 1:**
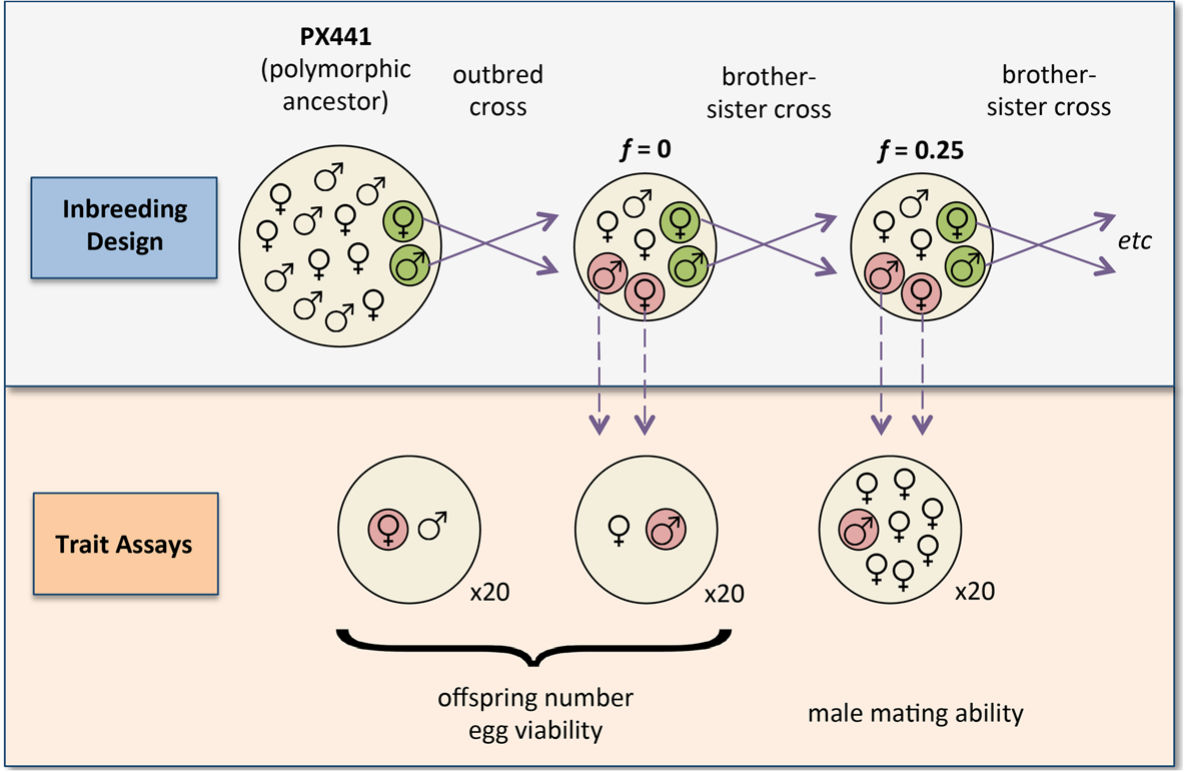
Summary of design of inbreeding and trait assays. Green circles illustrate worms that were inbred through five generations of brother-sister crosses, until *f* = 0.59. Red circles illustrate worms that were assayed each generation for three fitness-related traits. Only one of the ten total lineages is shown

The soil-dwelling nematode *C. remanei* provides an ideal model system to develop a framework for the causes and consequences of sex-specific inbreeding depression. *C. remanei* is comprised of equal numbers of XX females and hemizygous XO males and, as an obligate outcrosser, harbors high levels of polymorphism [31–34] and suffers dramatically from inbreeding depression [35]. The evolution of mating systems and sexual selection within *Caenorhabditis* is relatively well-understood [36–38], and the *C. remanei* transcriptome has been sequenced for males and females, indicating substantial gene expression differences between the sexes [39, 40]. Finally, while most previous work on sex-specific inbreeding depression has quantified fitness at one level of inbreeding, *C. remanei*’s 4-day generation time allows changes in fitness to be tracked over multiple generations, multiple levels of inbreeding, and multiple lineages. Our study examined 1400 males and females from nine inbred lineages over a range of inbreeding coefficients, tallying over 382,000 offspring (Fig. 1). The resulting patterns demonstrate that the genetic architecture underlying key fitness-related traits is largely sex-specific, and suggest that the pre- and post-mating costs of inbreeding depression may fall disproportionately on males and females.

## Methods

### Base population and maintenance conditions

The base population used for these inbreeding experiments was PX443, a highly polymorphic strain derived from the intercrossing of 26 isofemale lines isolated from wood lice in a patch of forest detritus near Toronto, Ontario (Koffler Scientific Reserve at Jokers Hill, King Township). The 26 isolates were crossed in a controlled fashion to promote equal contributions from all strains, including from mitochondrial genomes and X chromosomes [41]. Both the natural isolates and the resulting intercrossed population were frozen at −80 °C within 3 generations, and outbred PX443 was thawed periodically throughout the course of these experiments to minimize lab adaptation.

*C. remanei* were maintained under the standard laboratory conditions employed for culture of the closely related *C. elegans* [42]. Briefly, several large populations of PX443 (N ~ 10,000) were cultured for two generations to recover from freeze-thaw on 100 mm agar plates, seeded with *E. coli* strain OP50 as a food source. Crosses were performed on smaller, seeded 35 mm plates. Individual nematodes were manipulated and transferred from plate to plate with a fine “pick” made of platinum wire. All plates were maintained at 20 °C when in use, with some stored at 15 °C to slow development of offspring to be counted.

### Inbreeding design

To measure the fitness effects of inbreeding depression over time, nine independent lineages of PX433 were maintained through virgin brother-sister matings for five generations, with fitness-related traits of the inbred offspring assayed each generation (Fig. 1).

Ten lineages were initiated from independent crosses between a randomly chosen male and virgin female from PX443 (Fig. 1) in ten overlapping experiments between January and June, 2012. Offspring from these crosses were assumed to be completely outbred and were used in the *f* = 0 assays to establish baseline fitness for each lineage [43]. For each subsequent generation, five replicate pairs of one virgin sister and brother per lineage were crossed together, starting with the *f* = 0 brood. From the crosses that produced large enough brood sizes to assay, one brood was chosen at random to provide inbred worms to assay for fitness, as well as to continue inbreeding into the next generation with five new brother-sister pairs. Assays were performed after each generation of sibling mating, measuring the fitness of males and females at *f* = 0, 0.25, 0.38, 0.47, and 0.59. The second lineage failed to survive past the first sibling cross, when all five replicates produced no offspring, and so was not included in the subsequent analyses.

### Reproductive success assays

Reproductive success was measured by the number of offspring an inbred individual could produce after being housed with a single outbred mate for 24 h. This design allowed for multiple matings while minimizing the complex contributions of sexual conflict to lifetime reproductive success in *C. remanei* [44–46]. Because *C. remanei* tends to be sperm limited [45, 47], reproduction of inbred individuals with outbred individuals should reflect gamete number and quality, in addition to normal morphology and behavior, and provide a comparable measure of health and reproductive ability between males and females. Additionally, using an outbred partner for the inbred worm allowed us to determine its fecundity based only on its own level of inbreeding, without confounding effects of an inbred mate or inbred offspring.

For each value of *f*, 20 male and 20 female offspring from a brother-sister mating were picked to a total of 40 individual plates. Individuals were chosen on the cusp of sexual maturity (L4 final larval/young adult stage) to ensure virginity. Each worm was paired with an outbred PX443 opposite-sex partner for 24 h, after which period the male was removed. Every 24 h, the female was transferred to a fresh 35 mm plate. Each day’s offspring were counted separately upon reaching adulthood. When a female had ceased reproducing for two consecutive days, sperm were assumed to be depleted, and she was removed from the assay. A general linear model for offspring number was analyzed using JMP 9.0 (SAS Institute Inc., Cary, NC), with Lineage, Sex, and Sex*Lineage as categorical effects. Since each lineage was a random sample from the PX443 population, Lineage was treated as a random effect, leading to the Sex effect to be tested over the Sex*Lineage interaction. The effect of inbreeding (*f*) was estimated as a covariate, and the Sex*f term was used to estimate sex-specific responses to inbreeding depression.

### Survivorship assays

Normal, fertilized eggs laid during the 24 h that males and females were housed together for the reproductive success assays were used to determine egg-to-adult survivorship. For each sex and level of inbreeding, ten eggs were picked from 12 crosses and split among four plates of 30 eggs each to allow for an estimate of sampling error. After 4 days, once the surviving offspring reached adult stage, they were counted. Because the survivorship data consist of alive-dead counts, they were analyzed using a logistic regression with the same causal model as the reproductive assays.

### Mating assays

As a measure of male mating ability, one male was picked onto a 35-mm plate with seven adult virgin PX443 females for 1.5 h. Both males and females were isolated to sex-specific plates the previous day, while at L4 (final larval) stage, to ensure virginity. After 1.5 h, the male was removed, and the females were scored for signs of mating and removed to individual plates. As male *C. remanei* deposit copulatory plugs after successful sperm transfer [43], and sperm triggers the laying of eggs [48], females were considered mated if they bore a copulatory plug or laid normal, fertilized eggs. Because this is a male-specific effect, only the effects of Inbreeding and Lineage were fit in the linear model.

### Inbreeding depression (δ) and inbreeding load (*B*)

Inbreeding was estimated by first calculating the inbreeding load (*B*) via regression analysis and then using this value to calculate the level of inbreeding depression, δ [2, 4]. If deleterious mutations are largely independent of one another then they will act in a multiplicative manner, and the logarithm of mean fitness should decline in linear fashion as inbreeding increases [4]. Following this standard, inbreeding load (*B*) was calculated as the negative slope of the regression of the natural log of fitness (or relevant trait) on the inbreeding coefficient. Inbreeding depression (the relative strength of selection against an inbred genotype) was then calculated from *B* as:

1 $$ \updelta = 1\ \hbox{--}\ {\mathrm{e}}^{-BF} $$

*B* estimates the average number of lethal alleles per haploid genome (or per zygote if doubled) in the population under the assumption that recessive alleles are responsible for all of the deleterious effects of inbreeding [49], which recent evidence suggests is largely accurate [2, 50].

## Results

### Reproductive success

**Fig. 2:**
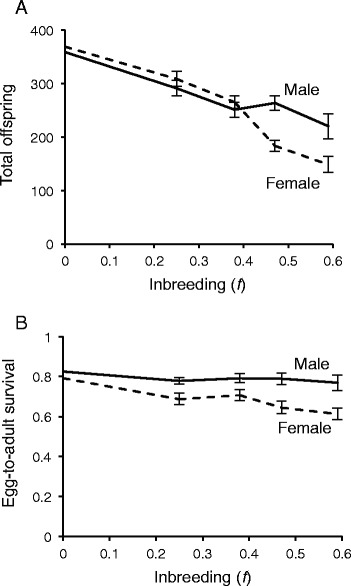
Sex-specific effects of inbreeding on (**a**) total offspring number and (**b**) egg-to-adult survival. Females experience more severe inbreeding depression than males in both traits. Error bars are one standard error

**Fig. 3:**
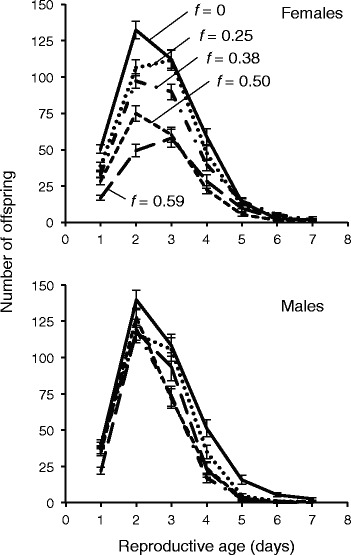
Age specific effects of inbreeding on reproductive output for females (top) and males (bottom). Day 1 indicates the first day of the cross

Inbreeding decreases reproductive success for *C. remanei* of both sexes, on average, but is significantly more harmful for females than males (Fig. 2a; Tables 2, 3). Inbreeding to *f* = 0.59 reduces brood sizes of females by an average of 57 % (206 offspring), compared to an average reduction of 37 % for males (128 offspring) (Table 3). While the reproductive output of both sexes declines equally after the first two generations of sibling mating, female brood size continues to decline after *f* = 0.38 while male brood size plateaus with subsequent inbreeding (Fig. 2a). When reproductive patterns are broken down by day, it is evident that peak reproduction declines with increasing levels of inbreeding in females, but not in males (Fig. 3). Consistent with this, there are twice as many lethal equivalents for offspring production for females than males (Table 3), and Sex *f has a significant effect in the linear model (*p* = 0.02; Table 2).

**Table 2:** Analysis of variance for the sex-specific effects of inbreeding on offspring number and survivorship

	Offspring number				Egg to adult viability		
	*df*	MS	*F*	*p*	*df*	χ^2^	*p*
Inbreeding (*f*)	1	123.62	37.38	<0.0001	1	246.9	<0.0001
Sex	1	5.86	0.58	0.4659	1	205.8	<0.0001
Lineage	8	59.17	5.80	0.0113	8	623.5	<0.0001
Sex x Lineage	8	10.20	3.08	0.0019	8	313.0	<0.0001
Sex x *f*	1	16.02	4.84	0.0279	1	44.9	<0.0001
Error	1,383	0.62			32,160		

Offspring analyzed via ANOVA on ln scale, viability via logistic regression

**Table 3:** Effects of inbreeding by trait and sex. All trait values are least squared mean estimates from the general linear model of nine lineages (±SEM)

Trait	Sex	*f* = 0	*f* = 0.59	δ	*B* (haploid)	*p-value*
Offspring number	♀	363.5 (±14.9)	157.8 (±18.6)	0.88	2.08 (±0.34)	<0.0001
	♂	348.1 (±14.9)	220.2 (±19.9)	0.62	0.98 (±0.37)	0.0081
Egg survivorship	♀	0.79 (±.037)	0.60 (±.040)	0.43	0.57 (±0.16)	0.0004
	♂	0.82 (±.037)	0.76 (±.041)	0.12	0.13 (±0.07)	0.0680
Mating ability	♂	2.00 (±.09)	1.68 (±0.12)	0.18	0.20 (±0.09)	0.0380

**Fig. 4:**
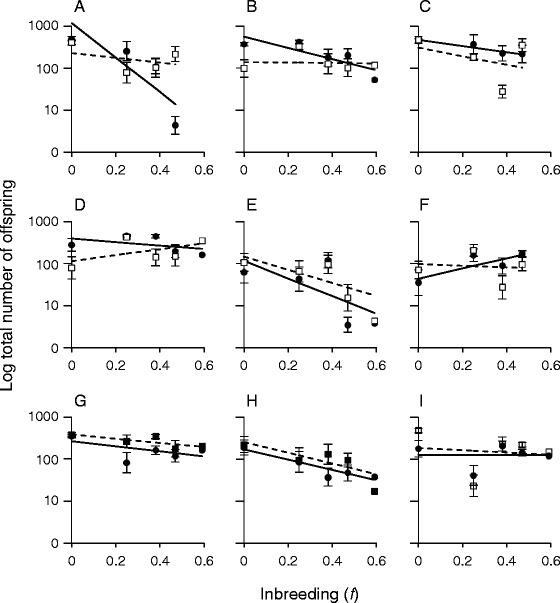
Lineage-specific regressions of male and female inbreeding depression on offspring number. Each plot (**a**-**i**) represents the inbreeding trajectory for one of the nine lineages analyzed in the experiment, in the order in which they were founded. Female values are represented by the dark circles and solid line; male values by the open squares and dashed line. On average, females lose fitness more quickly than males, although the actual responses are highly lineage-dependent

Six of the nine tested lineages suffered significant inbreeding depression when considered independently (Fig. 4). Of the three that did not, two lineages experienced no inbreeding depression for either sex (Fig. 4f,i), while the other lineage experienced inbreeding depression only for females (Fig. 4d). A total of eight lineages tolerated inbreeding to *f* = 0.59. One died out after *f* = 0.47, when visibly deformed male tails and protruding female vulvae made reproduction impossible (Fig. 4f).

### Egg to adult survivorship

Inbreeding significantly reduces the surviving proportion of a female’s eggs, but has no significant effect on the viability of eggs fathered by an inbred male (Fig. 2b, Tables 2, 3). At *f* = 0.59, the viability of female eggs is reduced by 24 %, versus 7 % for eggs sired by a comparably inbred male (Table 3). Inbreeding depression for this trait overall is less than that observed for offspring number, but because male inbreeding depression is not significantly different from zero (Table 3), Sex and Sex*f are both significant effects (Table 2).

### Male mating ability

**Fig. 5:**
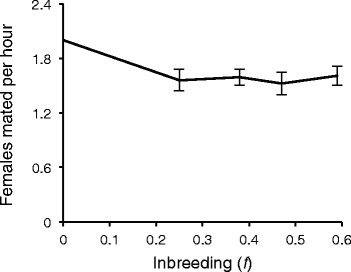
Average effect of inbreeding on male mating ability. Inbreeding depression appears to plateau after one generation of sibling mating. Error bars are one standard error

Male mating ability in a non-competitive environment declines significantly with inbreeding (Fig. 5; *F*_1,534_ = 4.95, *p* = 0.0266). However, the rate of decline is not uniform; after the first generation of sibling mating (*f* = 0.25), male mating ability is reduced by 35 %, but is not affected by further inbreeding (Fig. 5). The level of inbreeding depression for mating ability, as measured by δ, is only slightly greater than the male-specific estimate for egg-to-adult survivorship (Table 3). Notably, two of nine lineages actually experience outbreeding depression in this trait, though its magnitude is not significantly different from zero.

## Discussion

Sex-specific effects of inbreeding depression have been repeatedly identified in a range of organisms, but the direction and explanation of these effects have been inconsistent (Table 1). Our study found that *C. remanei* females suffer severe and continuous fitness declines with inbreeding, while males experience smaller losses that “plateau” beyond a moderate level of inbreeding (Figs. 2, 3, 5). Taken together, these results suggest that stronger selection in females is responsible for sex-specific inbreeding depression. This may be due both to the reproductive traits chosen, which are particularly influenced by loci involved in maternal effects, and to the influence of the X chromosome. However, it is critical to note that sexual selection, which typically places stronger pressure on males, was minimized in these experiments.

Females may experience stronger selection for loci involved in reproductive traits because of female-specific development and provisioning of eggs. The influence of maternal effects on offspring fitness is well-established [24–26] and has been invoked to explain female-specific inbreeding depression in a number of studies, particularly in birds [35, 51–54]. In this study, we observed inbreeding depression for egg survivorship only in females (Fig. 2b, Tables 2, 3), which supply eggs with the nutrients, energy, and specialized molecules necessary for development. In *C. elegans*, the genes coding for these yolk proteins are expressed solely in female tissues before being loaded into the egg [55]. It is then unsurprising that recessive mutations in genes affecting egg survivorship, which are made homozygous by inbreeding, have deleterious effects only in the sex in which they are expressed (Fig. 2b). Notably, based on the timing of the shift of transcriptional control from mother to zygote [56], this implies that most embryonic lethality from inbred females occurs early in development, prior to embryonic gastrulation.

Inbreeding depression for offspring number is likewise stronger in females at high levels of inbreeding. At earlier stages, fitness declines for males and females are statistically indistinguishable, suggesting that genes expressed in both sexes are involved in this trait (Fig. 2a, Table 2). However, the male fitness plateau after *f* = 0.38 implies that deleterious mutations expressed in males are exhausted at a lower level of inbreeding. This may be explained by differential target sizes for selection caused by some sex-specific expression, as for egg survivorship. Although *C. remanei* males tend to express more sex-specific transcripts than females overall [39, 40], egg viability and offspring number may be more dependent on female function than male function, implicating more female-specific genes. Understanding sex-specific patterns of expression for focal traits will be central to predicting sex-specific responses to inbreeding and their population-level consequences.

In addition to exhibiting less inbreeding depression in offspring production and egg survivorship, male *C. remanei* display relatively weak inbreeding depression in mating ability (Fig. 5, Table 3). This finding may be unique to a laboratory environment that minimizes competition and sexual selection by providing ample access to females. Stress and competition have repeatedly been shown to exacerbate inbreeding depression [6, 8, 16], particularly for male-male competition [57]. One key study found that inbred male mice have nearly normal fitness in laboratory conditions, but suffer severe inbreeding depression when housed in semi-natural enclosures requiring competition over resources, including mates [58]. Indeed, essentially all animal studies that have identified stronger inbreeding depression in males have attributed it to male-specific sexual selection (Table 1).

Because selection is generally expected to be stronger on males due to sexual selection ([27], but see also [59]), it is tempting to consider that reduced inbreeding depression in males could be a result of the purging of male-specific alleles through sexual selection. However, sexual selection influences many genes beyond those directly involved in male morphology and behavior: overall health and vigor is correlated with male success, and most mutations impacting health are likely to also impact male mating [55]. Consequently, mutations which are deleterious for health in both sexes may be purged more efficiently by sexual selection on males, in which they are more deleterious, than by natural selection on both sexes. This decrease in overall frequency, but maintenance of a larger selection coefficient in males, will actually reduce inbreeding depression to a greater degree for females than for males [16, 54, 55]. Thus, purging of deleterious alleles affecting male mating success in nature cannot explain the reduced inbreeding depression observed in males in this experiment. Instead, the lack of sexual selection in the laboratory likely reduces the selection coefficient for many deleterious alleles in males, leading to a relaxation of inbreeding depression.

Although the large declines in fitness observed in our experiment are consistent with empirical expectations for inbreeding depression [2], it is possible that some component of the laboratory environment could cause a systematic fitness decline in the tested lineages over time. In this case, we would be unable to separate environmental effects from the effect of inbreeding. However, the observed variation in fitness among inbred lines over the course of the experiment (Fig. 4) indicates that a systematic laboratory effect is unlikely, as lines respond differently at different points in time. Instead, our results are consistent with differential sampling of genetic variation from the ancestral population. Additionally, although our experiment necessarily excluded the most extreme effects of inbreeding by continuing lines with a minimum brood size, this design should not bias the results against females. Because inbreeding is more likely to reduce female than male brood sizes when comparing equally inbred brothers and sisters (Fig. 2a), the exclusion of small broods should disproportionately minimize the observed inbreeding depression for females, as compared to males. Overall, the inherent biology of mating and reproduction likely defines much of the sex-specific response to inbreeding, through sexual selection, maternal effects, or other sex-specific patterns of gene expression

Nonetheless, some proportion of the observed difference in sex-specific inbreeding depression could be explained by how male and female *C. remanei* differ in their chromosomal content: the X chromosome is present as a single copy in males (XO) and two copies in females (XX). Consequently, genes on the X are always effectively dominant in males, but may be recessive (and therefore contribute to inbreeding depression) in females. We found that females suffer 42 % more inbreeding depression in offspring number than males and 358 % more in egg viability, based on the calculated δ values (Fig. 2; Table 3). In comparison, 14 % of *C. elegans* genes are located on the X chromosome [39], and expression analysis in *C. elegans* has shown that the X is enriched for female-biased genes by a factor of about 1.4 [60]. Furthermore, in the absence of sexual selection, female-specific selection should dominate on the X chromosome, as demonstrated in a recent study on the evolution of sex chromosome dosage compensation [61]. Though effects on a trait need not be evenly distributed among genes, it seems likely that differential dominance on the X chromosome has some systematic consequence for sex-specific responses to inbreeding [50, 62]. However, differences of the magnitude observed here, especially for egg viability, may also require additional explanations, related to differential expression and selection.

Finally, the high level of variance in inbreeding depression observed in our study, while consistent with the high variance observed in many other studies (e.g. [7, 10, 11, 51, 52]), has particular bearing on the evolution of hermaphroditism in nematodes. The transition from gonochorism, characterized by outcrossing between males and females, to hermaphroditism, characterized by selfing, is thought to have occurred in nematodes at least ten times, compared to a single transition from hermaphroditism to gonochorism [63, 64]. For example, the common ancestor of *C. remanei* and the model hermaphrodite *C. elegans* was gonochoristic [65], but *C. elegans* has evolved to tolerate selfing rates upwards of 99 % [reviewed in 35] while *C. remanei* remains sensitive to inbreeding. However, the variation in this sensitivity suggests that hermaphroditism may be relatively common in nematodes in part because inbreeding depression is not a preventatively strong barrier. In two of our lineages, no significant inbreeding depression was detected at *f* = 0.59, and increasing levels of homozygosity actually had a positive (though non-significant) effect on the brood sizes of one sex in each lineage (Fig. 4). Dolgin et al. [35] similarly found that 5 of 39 *C. remanei* inbred lines survived 13 sequential generations of sibling mating, though they concluded that inbreeding depression could be a barrier to the evolution of hermaphroditism because the surviving lines were sickly. A recent RNAi study in *C. remanei* found that as few as two mutations, in the spermatogenesis and sperm activation pathways, can effectively change a female to a self-fertile hermaphrodite [66]. If such mutants happened to occur in genetic backgrounds with lesser degrees of inbreeding depression, as we observe here, then inbreeding depression need not be a strong barrier to the evolution of selfing. The combined low probability of the right mutations, in the right background, in the right ecological circumstance is likely to be the limiting factor governing such transitions in mating systems.

## Conclusions

Overall, the wide variety of results from sex-specific inbreeding studies appear to depend on the relative strengths of selection on males and females for the traits chosen in each experiment. For a given trait, inbreeding depression appears dependent on sex-specific expression of the relevant genes, their distribution on the sex chromosomes, and the environment in which the trait is measured, with particular emphasis on sexual selection. In the absence of sexual selection, we find that male *C. remanei* experience little to no inbreeding depression. However, female reproductive fitness declines steadily with inbreeding, suggesting that the post-mating mechanics of reproduction depend heavily on maternal effects. Predicting sex-specific responses to inbreeding, then, will require the identification of key traits and their sex-dependent expression in an appropriate environmental context. In particular, in light of the contribution of sexual selection to male inbreeding depression (e.g. [58]) minimizing sexual selection in males may help reduce inbreeding depression in threatened populations. Both to aid conservation, and to understand the consequences of inbreeding and mating system transitions in natural populations, it is important to recognize that inbreeding is affected by intrinsic biological differences between males and females, as they are filtered through the selective environment.

### Availability of supporting data

Data for this project is deposited in the Dryad data depository (http://datadryad.org) doi:10.5061/dryad.76rc3.
